# Sleep quality and its determinants in patients with thyroid eye disease: a cross-sectional study

**DOI:** 10.1186/s40001-025-02691-4

**Published:** 2025-06-03

**Authors:** Haiyan Chen, Jiamin Cao, Jiayang Yin, Bingxuan Wu, Min Zhu, Wei Xiong, Feng Zhang

**Affiliations:** 1 Department of Ophthalmology, Third Xiangya Hospital, Central South University, Yuelu District, 138 Tongzipo Road, Changsha, 410013 Hunan People’s Republic of China; 2 Wuzhou GongRen Hospital, No. 1, South Third Lane, Gaodi Road, Wuzhou City, 543001 Guangxi People’s Republic of China

**Keywords:** Thyroid eye disease, Sleep quality, Self-rating anxiety scale, Pittsburgh Sleep Quality Index

## Abstract

**Background:**

Sleep disorders may impair the nervous and immune systems. In thyroid eye disease (TED), an autoimmune condition, sleep quality and its potential determinants remain unclear. The study aimed to assess the sleep quality of patients with TED and identify its determinants.

**Method:**

A questionnaire was used to collect the clinical information of patients with TED and controls in a hospital. The data included the participants’ demographic characteristics, the Pittsburgh Sleep Quality Index (PSQI) score, and Self-rating Anxiety Scale (SAS) score. The sleep quality was compared between the TED and control groups. Furthermore, binary logistic regression analysis was used to detect the determinants of sleep quality, while linear regression was used to evaluate the relationships between PSQI and potential determinants.

**Results:**

Overall, 52 patients with TED and 75 healthy individuals were enrolled in this study. In the TED group, 59.6% of patients experienced sleep disorders, compared to only 33.3% in the control group. Additionally, the PSQI scores of patients with TED tended to be higher than those of controls (*p* = 0.002). Binary logistic regression revealed that a high SAS score was an independent risk factor for poorer sleep quality in patients with TED. Furthermore, patients with TED with higher GO-QoL visual function scores tended to have lower sleep scores.

**Conclusions:**

Patients with TED often experience sleep disorders, which are related to anxiety and visual impairment. Sleep disorders should be considered in managing patients with TED in clinical practice.

**Supplementary Information:**

The online version contains supplementary material available at 10.1186/s40001-025-02691-4.

## Background

Thyroid eye disease (TED), also referred to as thyroid-associated ophthalmopathy, is an autoimmune disorder characterized by extraocular muscle hypertrophy and orbital adipose tissue expansion [1]. The mechanical effects of proptosis and eyelid retraction disrupt the homeostasis of the ocular surface [2, 3], predisposing to pathological sequelae including dry eye syndrome [4–6] and corneal epithelial defects [7]. These structural abnormalities clinically manifest as persistent foreign body sensation and neuropathic ocular pain [8], both of which have been established as significant contributors to the deterioration of sleep quality [9–11]. Furthermore, elevated intraocular pressure observed in patients with TED [12] may compound these effects, given the established association between circadian variations in intraocular pressure and sleep pattern disruption demonstrated in previous ophthalmic research [13].

Patients with TED could have multidimensional psychosocial concerns [14], including cognitive deficits [15], anxiety, and depression [16]. These manifestations are corroborated by neuroimaging biomarkers [17] and large-scale epidemiological evidence [16]. Critically, psychological stress demonstrates bidirectional interactions with sleep impairment [18, 19]. Additionally, previous studies have revealed the relationships between thyroid dysfunction and sleep disturbances [20–24], with sleep improvement reported after normalization of thyroid hormone levels in patients with hyperthyroidism [25]. However, some data support that subclinical thyroid disorders are not significantly correlated with poor sleep quality [26].

Despite the clinical relevance of sleep quality in holistic disease management, the sleep quality of patients with TED and its determinants remains underexplored. Understanding these aspects is critical for improving the overall management and quality of life of patients with TED. Therefore, this study aims to investigate the sleep quality of patients with thyroid eye disease (TED) and its potential associated factors, hoping to use sleep quality as an entry point to aid in the diagnosis and management of TED patients.

## Methods

### Study design

This study adopted a cross-sectional observational design. It was conducted at the Third Xiangya Hospital of Central South University from February to September 2023, and patients with TED who met Bartley’s diagnostic criteria[27] and matched healthy controls were enrolled. The exclusion criteria included inability to complete questionnaires, major mental illness, or systemic conditions affecting sleep. Written informed consent was obtained from all participants. The study was approved by the Ethics Committee of the Third Xiangya Hospital.

Data collected included demographic characteristics (age, sex, education, marital status), TED-specific clinical parameters (proptosis, intraocular pressure, visual acuity, eyelid retraction, eyelid fissure width, clinical activity score (CAS) ≥3 for active disease), thyroid function (thyroid stimulating hormone (TSH), free T3/T4), quality of life (Graves’ Orbitopathy Quality of Life (GO-QoL)), sleep quality (Pittsburgh Sleep Quality Index (PSQI) total score >5), anxiety levels (Self-rating Anxiety Scale (SAS) standard score ≥50), and treatment history.

The primary objectives of this study were the prevalence and severity of sleep disorders in patients with TED. The secondary objectives included potential correlates of sleep disturbances, such as anxiety, GO-QoL, ocular parameter alterations (proptosis, intraocular pressure, visual acuity), thyroid dysfunction, and demographic characteristics.

### GO-QoL

The GO-QoL is the most thoroughly studied tool for evaluating the quality of life of patients with TED [28]. It includes eight questions on visual function and eight questions on self-assessment of appearance changes. Compared to merely observing ocular parameter alterations, this questionnaire places strong emphasis on addressing the psychological health concerns of patients with TED [29]. Notably, the characteristic emotional disturbances in patients with TED may be associated with sleep quality [30].

### PSQI

Sleep quality is the main research object of this study and was assessed using the PSQI. The PSQI assesses sleep quality in the past 6 months and includes seven factors, namely, subjective sleep quality, sleep latency, sleep duration, habitual sleep efficiency, sleep disorder, use of sleep drugs, and daytime dysfunction. The total score of 7 sub-projects is added up to obtain the total score. A total score of >5 indicates poor sleep quality [31].

### SAS

Anxiety is an important factor that affects sleep; therefore, symptoms of anxiety in patients with TED was assessed. The SAS is a 20-item rating scale that is used to assess symptoms of anxiety. Respondents were instructed to use a 4-point scale for evaluation, where 1 indicated “little or no time”, and 4 indicated “most of the time”. The total score for each volunteer was then calculated. The standard score was obtained by taking an integer after a rough score of 1.25; a higher score indicated greater anxiety. The abnormal threshold of the SAS standard score is 50 points, and more than 50 points can be regarded as anxiety [32].

### Statistical analysis

All statistical analyses were conducted using SPSS version 26.0 software (IBM Corp., Armonk, NY, USA). Demographic data were analyzed using frequencies and the Chi-squared test. Data conforming to a normal distribution were expressed using mean and standard deviation. The SAS scores and PSQI differences were compared between the two groups using the *T*-test. Pearson correlation or Spearman was used to explore the correlation between the main variables in the study. To eliminate the impact of different data units, all data were standardized before analysis. Binary logistic regression analysis was performed to identify factors that influence sleep quality.

## Results

### Demographic characteristics

A total of 127 participants (52 patients with TED and 75 normal controls) participated in a questionnaire survey after excluding missing or illogical variables. None of the participants had been administered psychotropic drugs or treatment in the past 3 months. Table 1 describes the basic characteristics of the participants. There was no statistically significant difference in sex distribution between the two groups (*p* > 0.05). More than half of the participants had completed their university education, which made it easier for them to understand the questionnaire. The proportion of participants with sleep disorders in the TED group was significantly higher (59.3%), than in the control group (33.3%; *p* < 0.05). Furthermore, the TED group had more participants with anxiety than the control group; however, the difference was not statistically significant (*p* > 0.05). Eye data of all participants with TED are shown in Table 2.

**Table 1:** Demography statistics and clinical information in the two groups

Category		TED (*n* = 52)	Normal control (*n* = 75)	χ^2^/*U*	*p*
Age	–	(30,48.75)	(26.35)	2701.5	0
Sex	Male	25(48.1%)	27(36.0%)	1.852	0.174
	Female	27 (51.9%)	48 (64.0%)		
Marital status	Single	8 (15.4)	23 (30.7%)	3.887	0.049
	Married	44 (84.6%)	52 (69.3%)		
Education	Junior middle education	9 (17.3%)	4 (5.3%)	8.338	0.015
	High school	9 (17.3%)	6 (8.0%)		
	University	34 (65.4%)	65 (86.7%)		
Sleep quality	Poor (global PSQI > 5)	31 (59.6%)	25 (33.3%)	8.605	0.003**
SAS state	SAS scores (>50)	17 (32.7%)	20 (26.7%)	0.54	0.462

*TED* thyroid-associated disease

**p-value <0.01 is considered to indicate a significant correlation (TED vs. normal control). PSQI, Pittsburgh Sleep Quality Index

**Table 2:** TED-specific parameters of all TED patients

Clinical information	TED (*N* = 52)
IOP in the worst eye (mmHg)	(18, 23)
Proptosis in the worst eye (mm)	17.87 ± 3.25
Vision in the worst eye (Log MAR)	(0, 0.21)
Have diplopia	13 (25.0%)
CAS score in the worst eye	(0, 3)
*Severity in the worst eye*	
Mild (*n*, %)	4 (7.7%)
Moderate-to-severe (*n*, %)	37 (71.2%)
Sight-threatening (*n*, %)	11 (21.2%)
Duration of thyroid dysfunction (month)	(7.5, 45.0)
Duration of TED (month)	(5.0, 36.0)
GO-QoL-visual scores	(37.50, 75.00)
GO-QoL-psychosocial scores	(31.25, 54.69)
Euthyroid	46 (88.46%)
Hyperthyroidism	3 (5.77%)
Hypothyroidism	3 (5.77%)

*IOP* intraocular pressure

### TED had a higher ratio of sleep disorders compared with controls

Compared with the control group, the sleep quality of the TED group was generally poor (*p* < 0.05), mainly reflecting their subjective sleep scores, sleep latency, habitual sleep efficiency, and daytime dysfunction (Table 3). Patients with thyroid dysfunction are prone to mood and anxiety disorders [33]; our results showed that the anxiety score of the TED group was slightly higher than that of the normal group, but the difference was not statistically significant (*p* > 0.05).

**Table 3:** Comparison of sub-scores and total scores of the PSQI scale between the TED group and control group

	TED group (*n* = 52)	Control group (*n* = 75)	*F*	*p* value
Subjective sleep score	1.38 ± 0.867	0.73 ± 0.704	2.856	0.000**
Sleep latency	1.73 ± 0.910	1.19 ± 0.766	5.295	0.001**
Sleep duration	0.44 ± 0.802	0.55 ± 0.664	0.162	0.426
Habitual sleep efficiency	0.75 ± 1.007	0.31 ± 0.636	17.713	0.006*
Sleep disturbance	1.23 ± 0.581	1.16 ± 0.616	0.000	0.516
Use of sleep medications	0.23 ± 0.645	0.32 ± 0.596	1.302	0.424
Daytime dysfunction	1.25 ± 1.100	0.75 ± 0.840	5.266	0.007*
Global PSQI	7.02 ± 3.812	5.00 ± 3.468	0.058	0.002*
SAS	44.19 ± 10.531	43.12 ± 12.472	2.501	0.613

Note: All data are represented by mean ± SD

*p-value <0.05

**p-value <0.01. PSQI, Pittsburgh Sleep Quality Index

### SAS total score and GO-QOL-visual scores were associated with sleep quality of TED

**Fig. 1:**
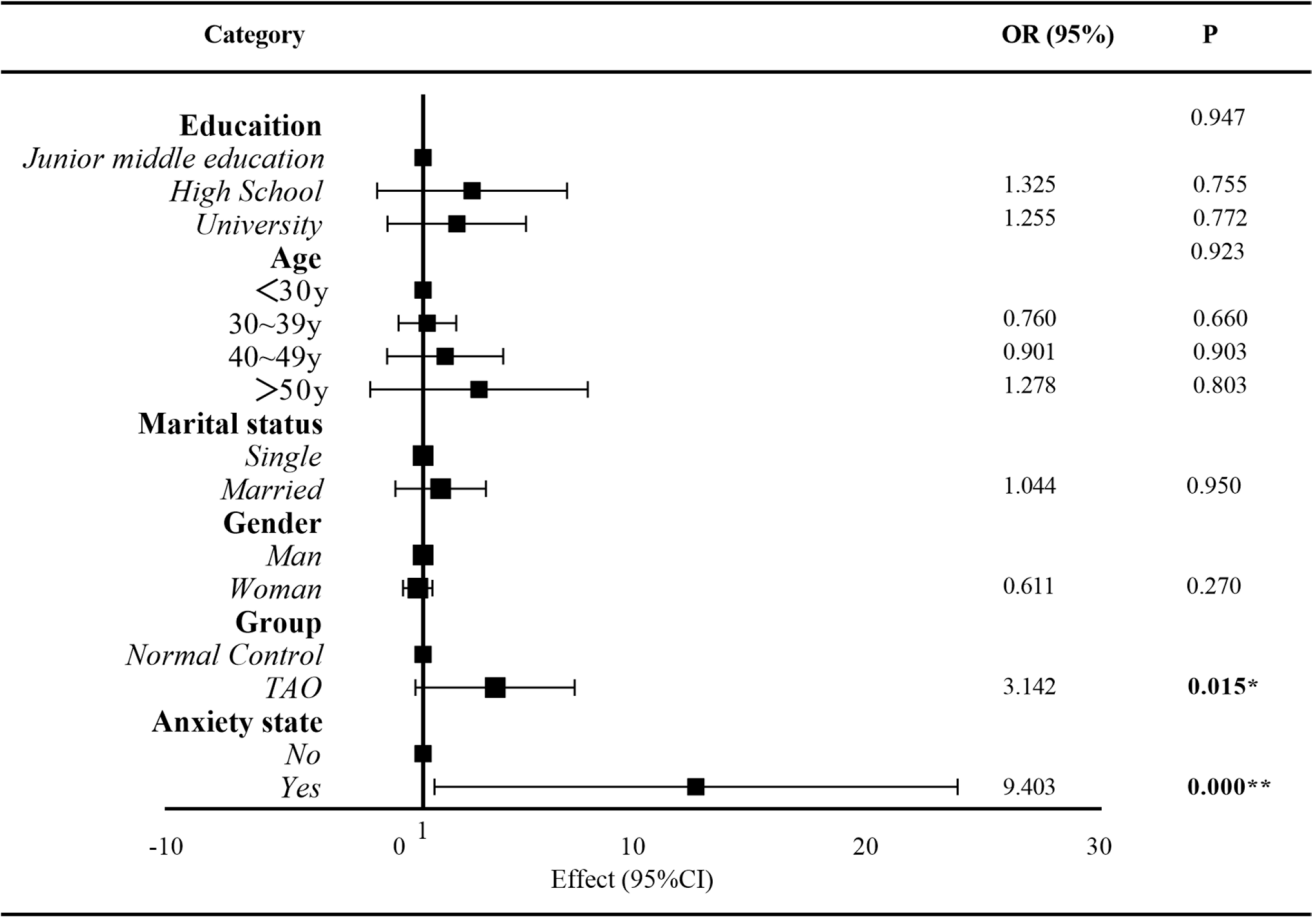
Binary logistic regression of risk factors for all participants

Binary logistic regression analysis revealed that TED and anxiety were risk factors that affect sleep quality (Fig. 1). The sleep quality of patients in the TED group was further analyzed using univariate analysis of variance. It was discovered that sex, worsening eye protrusion, SAS score, diplopia, GO-QoL visual function, and GO-QoL social function were all correlated. Binary logistic regression results of sleep quality in patients with TED suggest that GO-QoL-visual function (OR = 0.97, 95% CI 0.94–0.99; *p* = 0.018) and SAS total score (OR = 1.13, 95% CI 1.04–1.23; *p* = 0.006) are independent predictive indicators for poor sleep quality in patients with TED (Table 4).

**Table 4:** Binary logistic regression analysis of the sleep quality of patients with TED

	Beta	SE	*p*	Exp (B)	95% CI
SAS scores	0.123	0.045	0.006**	1.131	(1.036, 1.234)
GO-QoL-visual scores	−0.035	0.045	0.018*	0.965	(0.938, 0.994)

^*^p<0.05; ^**^p<0.01

### Relationships between sleep quality and determinants

**Fig. 2:**
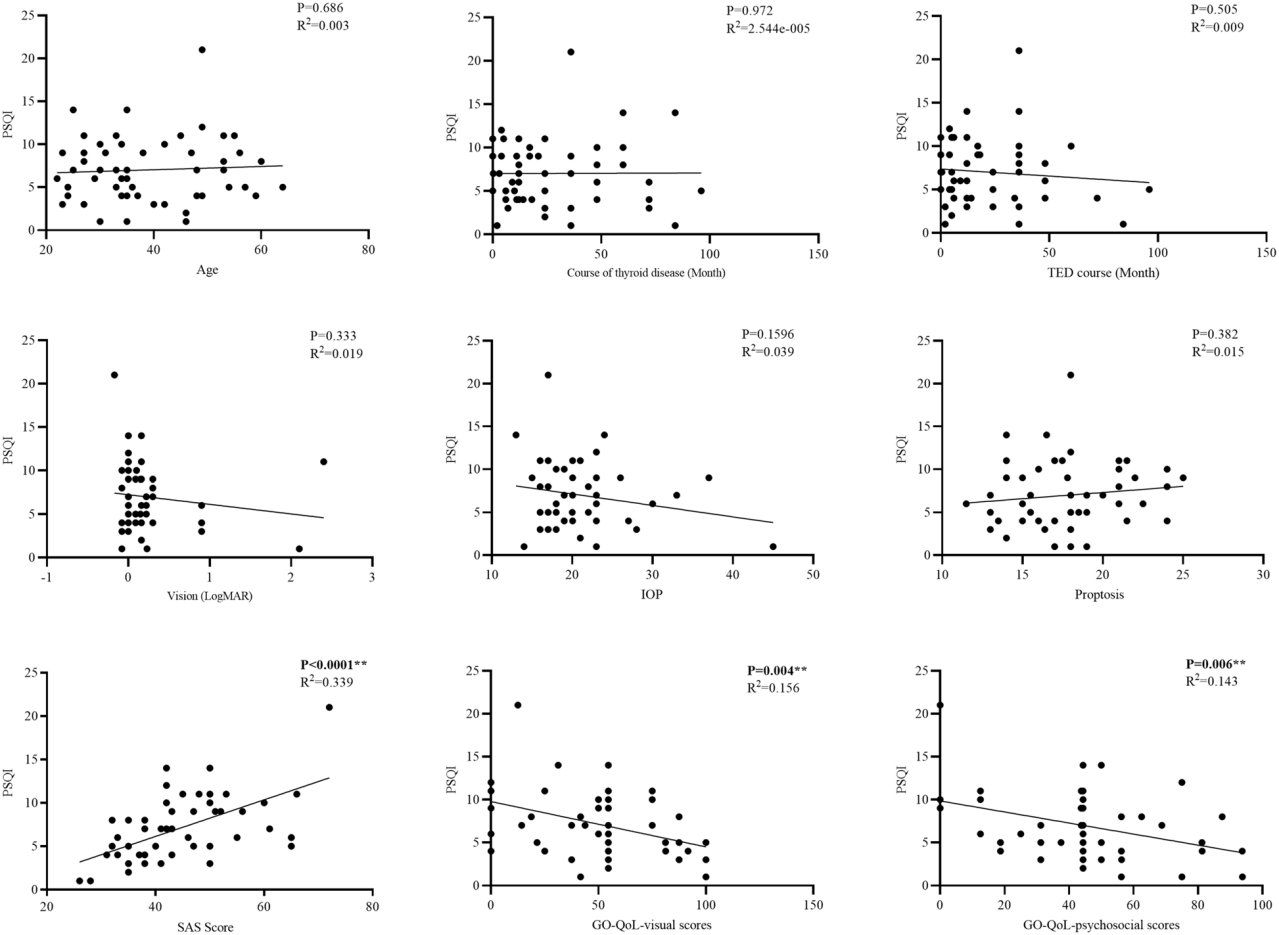
Correlation analysis of PSQI with potential associated factors

Linear regression analysis was used to identify the determinants of sleep quality. The result showed that SAS was positively correlated with sleep quality in patients with TED (Fig. 2). The more anxious patients are, the poorer their sleep quality (*p* < 0.001). Furthermore, the GO-QoL visual function score and GO-QoL social function score are negatively correlated with PSQI (*p* < 0.001). Given the potential impact of thyroid function on sleep quality, PSQI scores were analyzed in patients with TED who had varying thyroid functions. After subdividing patients with TED into different groups based on their thyroid function status (euthyroid, hyperthyroidism, and hyperthyroidism groups), no significant differences in total PSQI scores were observed across the various thyroid function states (*p* = 0.39). Additionally, there was no significant correlation between the thyroid function and SAS total score of patients with TED (*p* > 0.05).

## Discussion

This study delved into sleep quality and its determinants in patients with TED. We found that approximately 59.6% of patients with TED experienced sleep disorders. Sleep disorders have been commonly described in other ocular diseases, such as dry eye syndrome, glaucoma, and fundus changes, while the association between TED and sleep quality has been rarely addressed [34]. Our analysis indicated that anxiety and diminished visual function were associated with sleep disorders in patients with TED. However, no statistically significant correlation was observed among visual acuity, intraocular pressure, proptosis, disease duration, and age.

Our findings indicate that anxiety may be associated with poor sleep quality in patients with TED. Previous studies have also demonstrated an association between anxiety and sleep disorders [35]. Additionally, functional magnetic resonance imaging (fMRI) has revealed overlapping brain networks involved in sleep and emotional regulation [36]. Furthermore, Xu et al. used brain imaging evidence to demonstrate a significant correlation between the severity of insomnia and anxiety in patients [37]. While observational data suggest a sleep–anxiety association, causal inference requires validation through randomized controlled trials or Mendelian randomization.

Correlation analysis revealed that lower GO-QoL visual function scores were associated with poorer sleep quality in patients with TED. Furthermore, a statistically significant inverse correlation was observed between the GO-QoL visual score and sleep quality score, indicating that higher visual function was associated with poorer sleep quality. These findings suggest a potential association between visual function, social function, and sleep quality in patients with thyroid-related ophthalmopathy.

Light affects the regulation of the circadian rhythms. If light signals cannot reach the hypothalamic circadian clock, disorders of the circadian rhythm may occur [38]. Patients with TED may have problems such as decreased visual acuity, diplopia, and reduced visual quality, which can lead to the transmission of abnormal light signals and may cause circadian rhythm disorders and sleep disturbances.

Emerging evidence highlights that thyroid dysfunction is frequently associated with comorbid anxiety in patients [39], thereby contributing to impaired sleep quality [40]. Hyperthyroidism-induced stimulation of the sympathetic nerves can lead to challenges in falling and staying asleep, along with experiencing daytime lethargy [20]. Thyroid function normalization following treatment can alleviate subjective sleep disturbances caused by increased sympathetic nervous system tone in hyperthyroid patients [25]. In addition to other factors, insufficient sleep can lead to an increased sympathetic tone [41]. Notably, our study found no significant differences in sleep quality among patients with varying thyroid function states. This may be because most of the participants had well-controlled thyroid function. Future research should stratify participants according to their thyroid function status to explore this relationship.

This study has some limitations. First, it is a cross-sectional study; therefore, the causal relationship between sleep and its determinants could not be examined. Second, the sleep quality questionnaire is subjective and may not accurately reflect the actual sleep quality [42]. Polysomnography may offer a more accurate detection method for sleep quality. Analysis through objective detection methods makes the assessment of sleep quality in patients with TED more convincing.

## Conclusions

Patients with TED may have sleep disorders, which may be related to anxiety, thyroid function, and visual impairment. Understanding and addressing sleep disorders in patients with TED may be beneficial in improving the treatment efficacy and prognosis of TED.

## Supplementary Information

Additional file 1.

## Abbreviations

TED: Thyroid eye disease
PSQI: Pittsburgh Sleep Quality Index
CAS: Clinical activity score
SAS: Self-rating Anxiety Scale
GO-QoL: Graves’ Orbitopathy Quality of Life
IOP: Intraocular pressure
TSH: Thyroid stimulating hormone

## Declarations

### Availability of data and materials

The datasets used and/or analyzed during the current study are available from the corresponding author on reasonable request.

## References

[1] Łacheta D, Miśkiewicz P, Głuszko A, Nowicka G, Struga M, Kantor I, et al. Immunological aspects of Graves’ ophthalmopathy. Biomed Res Int. 2019;2019:7453260. 31781640 10.1155/2019/7453260 PMC6875285

[2] Rana HS, Akella SS, Clabeaux CE, Skurski ZP, Aakalu VK. Ocular surface disease in thyroid eye disease: A narrative review. Ocul Surf. 2022;24:67–73. 35167950 10.1016/j.jtos.2022.02.001 PMC9058200

[3] Yu Y, Hu YX, Lu MX, Ouyang ZL, Xu MT, Zhao LY, et al. Risk factors for ocular surface irritation symptoms in inactive mild and moderate-to-severe Graves’ orbitopathy. Ophthalmol Ther. 2024;13(4):1015–24. 38376797 10.1007/s40123-024-00892-4 PMC10912376

[4] Park J, Baek S. Dry eye syndrome in thyroid eye disease patients: The role of increased incomplete blinking and Meibomian gland loss. Acta Ophthalmol. 2019;97(5):e800–6. 30593716 10.1111/aos.14000

[5] Riguetto CM, Barbosa EB, Atihe CC, Reis F, Alves M, Zantut-Wittmann DE. Ocular surface disease related to the inflammatory and non-inflammatory phases of thyroid eye disease. Clin Ophthalmol. 2023;17:3465–75. 38026592 10.2147/OPTH.S430861 PMC10657741

[6] Sun R, Yang M, Lin C, Wu Y, Sun J, Zhou H. A clinical study of topical treatment for thyroid-associated ophthalmopathy with dry eye syndrome. BMC Ophthalmol. 2023;23(1):72. 36803227 10.1186/s12886-023-02805-8 PMC9940084

[7] Lai EW, Tai YH, Wu HL, Dai YX, Chen TJ, Cherng YG, et al. The Association between autoimmune thyroid disease and ocular surface damage: a retrospective population-based cohort study. J Clin Med. 2023;12(9):3203. 37176642 10.3390/jcm12093203 PMC10179488

[8] Cockerham KP, Padnick-Silver L, Stuertz N, Francis-Sedlak M, Holt RJ. Quality of life in patients with chronic thyroid eye disease in the United States. Ophthalmol Ther. 2021;10(4):975–87. 34478126 10.1007/s40123-021-00385-8 PMC8589903

[9] Ayaki M, Kawashima M, Negishi K, Kishimoto T, Mimura M, Tsubota K. Sleep and mood disorders in dry eye disease and allied irritating ocular diseases. Sci Rep. 2016;6:22480. 26927330 10.1038/srep22480 PMC4772386

[10] Magno MS, Utheim TP, Snieder H, Hammond CJ, Vehof J. The relationship between dry eye and sleep quality. Ocul Surf. 2021;20:13–9. 33421635 10.1016/j.jtos.2020.12.009

[11] Wu M, Liu X, Han J, Shao T, Wang Y. Association between sleep quality, mood status, and ocular surface characteristics in patients with dry eye disease. Cornea. 2019;38(3):311–7. 30614900 10.1097/ICO.0000000000001854

[12] Fan SX, Zeng P, Li ZJ, Wang J, Liang JQ, Liao YR, et al. The clinical features of Graves’ orbitopathy with elevated intraocular pressure. J Ophthalmol. 2021;2021:9879503. 33564472 10.1155/2021/9879503 PMC7850838

[13] Aptel F, Canaud P, Tamisier R, Pépin JL, Mottet B, Hubanova R, et al. Relationship between nocturnal intraocular pressure variations and sleep macrostructure. Invest Ophthalmol Vis Sci. 2015;56(11):6899–905. 26505463 10.1167/iovs.15-17456

[14] Kahaly GJ, Petrak F, Hardt J, Pitz S, Egle UT. Psychosocial morbidity of Graves’ orbitopathy. Clin Endocrinol (Oxf). 2005;63(4):395–402. 16181231 10.1111/j.1365-2265.2005.02352.x

[15] Bruscolini A, Iannitelli A, Segatto M, Rosso P, Fico E, Buonfiglio M, et al. Psycho-cognitive profile and NGF and BDNF levels in tears and serum: a pilot study in patients with Graves’ disease. Int J Mol Sci. 2023;24(9):8074. 37175781 10.3390/ijms24098074 PMC10178719

[16] Lee TC, Radha-Saseendrakumar B, Delavar A, Ye GY, Ting MA, Topilow NJ, et al. Evaluation of depression and anxiety in a diverse population with thyroid eye disease using the nationwide NIH all of us database. Ophthalmic Plast Reconstr Surg. 2023;39(3):281–7. 36727790 10.1097/IOP.0000000000002318

[17] Luo L, Gao L, Li D, Wen H. Depression- and anxiety-associated disrupted brain structural networks revealed by probabilistic tractography in thyroid associated ophthalmopathy. J Affect Disord. 2024;347:515–25. 38042306 10.1016/j.jad.2023.11.089

[18] Akerstedt T. Psychosocial stress and impaired sleep. Scand J Work Environ Health. 2006;32(6):493–501. 17173205

[19] LeBlanc M, Mérette C, Savard J, Ivers H, Baillargeon L, Morin CM. Incidence and risk factors of insomnia in a population-based sample. Sleep. 2009;32(8):1027–37. 19725254 10.1093/sleep/32.8.1027 PMC2717193

[20] Sridhar GR, Putcha V, Lakshmi G. Sleep in thyrotoxicosis. Indian J Endocrinol Metab. 2011;15(1):23–6. 21584162 10.4103/2230-8210.77578 PMC3079865

[21] Xia L, Chen GH, Li ZH, Jiang S, Shen J. Alterations in hypothalamus-pituitary-adrenal/thyroid axes and gonadotropin-releasing hormone in the patients with primary insomnia: a clinical research. PLoS One. 2013;8(8): e71065. 23951080 10.1371/journal.pone.0071065 PMC3739817

[22] Addanki S, Patel K, Patel L, Smith B, Patel P, Uppalapati S, et al. Thyroid function and sleep patterns: a systematic review. Cureus. 2024;16(6): e63447. 39077291 10.7759/cureus.63447 PMC11285688

[23] Song L, Lei J, Jiang K, Lei Y, Tang Y, Zhu J, et al. The association between subclinical hypothyroidism and sleep quality: a population-based study. Risk Manag Healthc Policy. 2019;12:369–74. 31908553 10.2147/RMHP.S234552 PMC6927586

[24] Alreshidi NF, Alenzi H, Alrashidi R, Aljaloud LZ, Alshammari AB. The relationship between thyroid dysfunction and sleep quality among population of Saudi Arabia. Int J Gen Med. 2024;17:2497–505. 38831928 10.2147/IJGM.S462512 PMC11144793

[25] Matsumoto K, Izawa S, Fukaya K, Matsuda E, Fujiyama M, Matsuzawa K, et al. Hyperthyroidism in Graves disease causes sleep disorders related to sympathetic hypertonia. J Clin Endocrinol Metab. 2022;107(5):e1938–45. 35022743 10.1210/clinem/dgac013

[26] Akatsu H, Ewing SK, Stefanick ML, Fink HA, Stone KL, Barrett-Connor E, et al. Association between thyroid function and objective and subjective sleep quality in older men: the osteoporotic fractures in men (MrOS) study. Endocr Pract. 2014;20(6):576–86. 24449663 10.4158/EP13282.OR PMC4363141

[27] Bartley GB, Gorman CA. Diagnostic criteria for Graves’ ophthalmopathy. Am J Ophthalmol. 1995;119(6):792–5. 7785696 10.1016/s0002-9394(14)72787-4

[28] Lee THB, Sundar G. Quality of life in thyroid eye disease: a systematic review. Ophthalmic Plast Reconstr Surg. 2020;36(2):118–26. 31567783 10.1097/IOP.0000000000001446

[29] Wiersinga WM. Quality of life in Graves’ ophthalmopathy. Best Pract Res Clin Endocrinol Metab. 2012;26(3):359–70. 22632371 10.1016/j.beem.2011.11.001

[30] Pearson O, Uglik-Marucha N, Miskowiak KW, Cairney SA, Rosenzweig I, Young AH, et al. The relationship between sleep disturbance and cognitive impairment in mood disorders: A systematic review. J Affect Disord. 2023;327:207–16. 36739007 10.1016/j.jad.2023.01.114

[31] Buysse DJ, Reynolds CF 3rd, Monk TH, Berman SR, Kupfer DJ. The Pittsburgh Sleep Quality Index: a new instrument for psychiatric practice and research. Psychiatry Res. 1989;28(2):193–213. 2748771 10.1016/0165-1781(89)90047-4

[32] Dunstan DA, Scott N. Norms for Zung’s Self-rating Anxiety Scale. BMC Psychiatry. 2020;20(1):90. 32111187 10.1186/s12888-019-2427-6 PMC7048044

[33] Bunevicius R, Velickiene D, Prange AJ Jr. Mood and anxiety disorders in women with treated hyperthyroidism and ophthalmopathy caused by Graves’ disease. Gen Hosp Psychiatry. 2005;27(2):133–9. 15763125 10.1016/j.genhosppsych.2004.10.002

[34] Lee SSY, Nilagiri VK, Mackey DA. Sleep and eye disease: A review. Clin Exp Ophthalmol. 2022;50(3):334–44. 35263016 10.1111/ceo.14071 PMC9544516

[35] Cox RC, Olatunji BO. A systematic review of sleep disturbance in anxiety and related disorders. J Anxiety Disord. 2016;37:104–29. 26745517 10.1016/j.janxdis.2015.12.001

[36] Chellappa SL, Aeschbach D. Sleep and anxiety: From mechanisms to interventions. Sleep Med Rev. 2022;61: 101583. 34979437 10.1016/j.smrv.2021.101583

[37] Xu M, Li B, Wang S, Chen C, Liu Z, Ji Y, et al. The brain in chronic insomnia and anxiety disorder: a combined structural and functional fMRI study. Front Psychiatry. 2024;15:1364713. 38895035 10.3389/fpsyt.2024.1364713 PMC11184054

[38] Lockley SW, Arendt J, Skene DJ. Visual impairment and circadian rhythm disorders. Dialogues Clin Neurosci. 2007;9(3):301–14. 17969867 10.31887/DCNS.2007.9.3/slockley PMC3202494

[39] Inman BL, Long B. Thyrotoxicosis. Emerg Med Clin North Am. 2023;41(4):759–74. 37758422 10.1016/j.emc.2023.06.005

[40] Rehman UL, Khalid M, Fatima M, Khan MS, Abro MT, Waafira A. Anxiety and depression among adolescents and young adults with thyroid function disorders: a cross-sectional study. Expert Rev Endocrinol Metab. 2025. 10.1080/17446651.2025.2480693. 40108945 10.1080/17446651.2025.2480693

[41] Seravalle G, Mancia G, Grassi G. Sympathetic nervous system, sleep, and hypertension. Curr Hypertens Rep. 2018;20(9):74. 29980938 10.1007/s11906-018-0874-y

[42] Moon HJ, Song ML, Cho YW. Clinical characteristics of primary insomniacs with sleep-state misperception. J Clin Neurol. 2015;11(4):358–63. 26256663 10.3988/jcn.2015.11.4.358 PMC4596102

