# Supplementary material for: Sleep quality and its determinants in patients with thyroid eye disease: a cross-sectional study
Source: Eur J Med Res. 2025 Jun 3;30:446. doi: 10.1186/s40001-025-02691-4 (PMC12131440; doi:10.1186/s40001-025-02691-4)
Supplement: Supplementary file 1 — Additional file 1. [file 40001_2025_2691_MOESM1_ESM.docx]

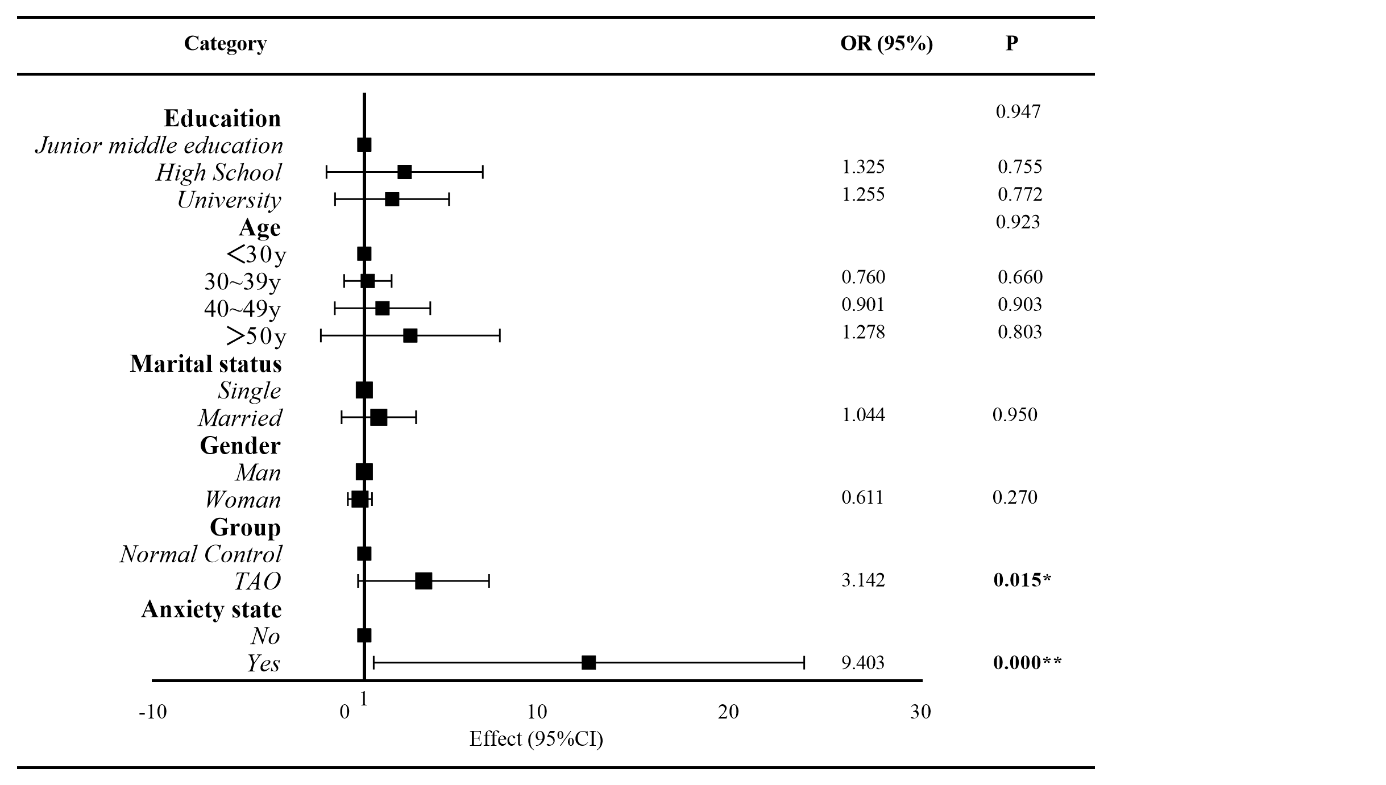


**Figure 1.** Binary logistic regression of risk factors for all participants.


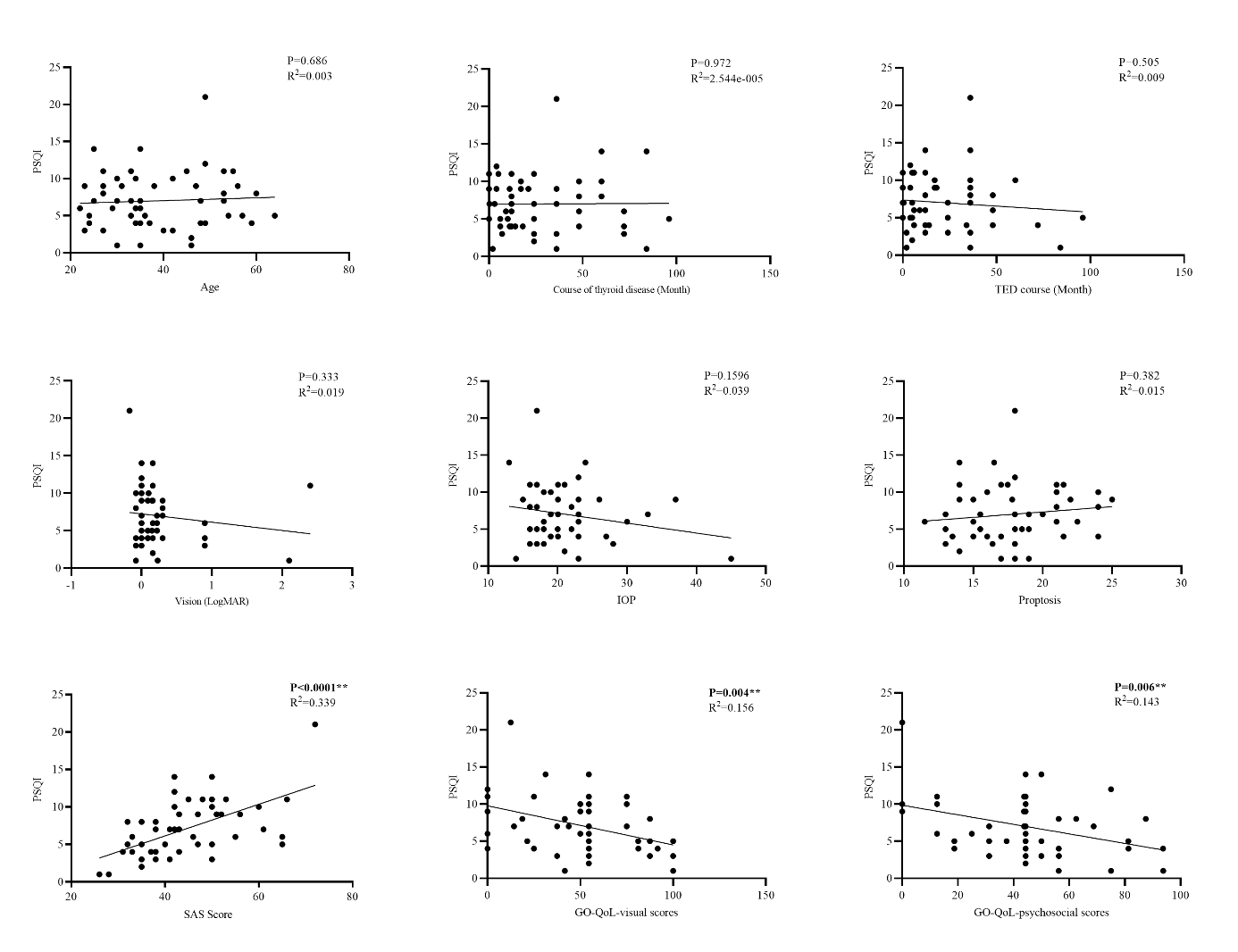


**Figure 2.** Correlation analysis of PSQI with potential associated factors.
